# AI and the transformation of clinical reasoning: A classroom perspective

**DOI:** 10.1371/journal.pdig.0001478

**Published:** 2026-06-04

**Authors:** Nghia Phu Nguyen

**Affiliations:** 1 College‌‌ of Health Sciences, Nam Can Tho University, Can Tho City, Vietnam; 2 Cardiovascular Research Department, Methodist Hospital, Merrillville, Indiana, United States of America; Shahid Beheshti University of Medical Sciences School of Dentistry, IRAN, ISLAMIC REPUBLIC OF

Artificial intelligence (AI) is rapidly changing how medicine is taught and learned. Evidences show that AI can support problem-based learning (PBL) and case-based learning (CBL), improving knowledge acquisition by up to 46%, increasing learner satisfaction, and enhancing clinical reasoning and clinical competence when integrated into interactive learning environments with real-time feedback [1,2]. AI-integrated training programs have demonstrated superior learning performance [3]. Tools such as virtual patient simulations and real-time feedback systems not only enhance diagnostic reasoning but also facilitate self-directed learning by providing immediate, adaptive feedback and personalize the learning experience [3,4]. Moreover, AI supports curriculum innovation through automated assessment and data analytics, enabling medical students to better adapt to the demands of modern healthcare systems [3,4].

Yet, in the reality of classrooms, a quieter disruption is unfolding. Medical education relies on multiple complementary approaches—including simulation-based training, team-based learning (TBL), bedside teaching, and CBL—all of which are potentially vulnerable to uncritical AI use. In CBL sessions, medical students increasingly turn to large language models (LLMs) to answer guiding questions, raising concerns about reduced engagement in clinical reasoning processes despite improvements in efficiency and short-term performance [1,2]. In TBL, reliance on AI-generated answers may diminish peer discussion, accountability, and collective problem-solving, potentially leading to reduced cognitive engagement and increased overreliance in AI-assisted learning environments [5,6]. In simulation-based training, AI-supported systems can enhance realism and efficiency, but their impact on deeper clinical reasoning and independent decision-making remains uncertain, particularly in terms of long-term retention and transfer to real clinical practice [3]. Even in bedside teaching, overdependence on AI tools may shift attention away from direct patient interaction and clinical observation, potentially weakening experiential learning and independent clinical judgment. Across these educational contexts, processes intended to stimulate active reasoning and engagement risk being reduced to the passive reproduction of AI-generated responses. From an educational perspective, this shift may threaten the development of clinical reasoning in classroom settings, where learning is meant to occur through dialogue, uncertainty, and active cognitive engagement.

The flow of clinical reasoning in classroom-based learning typically unfolds through structured prompts, such as: *What is the most likely diagnosis? What are the differential diagnoses? What is the next step in management? Which investigations are warranted to confirm the diagnosis? How should these findings be interpreted?.* These questions are meant to spark hypothesis generation, evidence weighing, and peer critique. However, students can now easily bypass this process by simply pasting a question into LLMs, obtaining a polished answer within seconds. Students may lose the struggle that builds diagnostic judgment, instead memorizing AI-provided statements [5]. Group dynamics also suffer: if one student supplies an AI-generated answer, peers are less inclined to debate. What was intended as co-construction of knowledge becomes passive acceptance. Educators, meanwhile, struggle to discern whether reasoning is genuine or AI-generated [7]. At the same time, educators face a growing adaptation gap, as students often adopt AI rapidly through informal use while faculty remain more cautious and dependent on institutional guidance, making it increasingly difficult to effectively supervise and align learning practices [8]. This mismatch may widen the gap between teaching and learning processes, limiting educators’ ability to effectively guide critical reasoning and increasing the risk that students’ AI use remains insufficiently supervised or critically examined.

Recent evidence underscores the concern. AI-augmented PBL improved immediate test scores but relied on a small number of trials at high risk of bias [1]. Studies of ChatGPT-assisted CBL show efficiency in case creation but uncertain impact on deeper reasoning [2,9]. Evidences reveal that while students are generally enthusiastic about AI use, they also express concerns regarding ethical and fairness issues, including potential algorithmic bias, risks to data privacy, academic integrity, and the reliability of AI-generated information [4,7]. AI-generated clinical suggestions may reflect biases embedded in training data, which can limit generalizability and lead to poorer performance for underrepresented populations [10]. In a study, hallucinations—plausible but incorrect outputs—were common in complex clinical scenarios, with rates up to 69.6% and accuracy dropping to 44.2%, while trainees detected only about 55% of these errors [11]. This suggests that trainees may struggle to critically appraise AI-generated outputs, increasing the likelihood of accepting inaccurate reasoning or missing important differential diagnoses.

A natural reaction is prohibition: some schools discourage or ban AI use [7]. But such measures are impractical and shortsighted. Students can access AI on their phones regardless of policy. Guidance from the Association of American Medical Colleges (AAMC) instead emphasizes responsible and transparent integration of AI, outlining principles such as maintaining human-centered judgment, ensuring ethical use, and investing in faculty development rather than restricting access [12]. More importantly, future clinicians will inevitably work alongside AI systems; education should prepare them for that reality, not deny it [13]. This trajectory is already visible in practice: for example, Mass General Brigham has implemented an AI-supported primary care program that uses an AI agent to collect patient information and generate preliminary clinical summaries for physician review [14], while in Utah, a state-approved pilot program allows the AI system to autonomously renew prescriptions for selected chronic medications under defined safeguards [15]. In a recent study, the LLM-based conversational AI system Articulate Medical Intelligence Explorer (AMIE) was prospectively evaluated at an academic urgent care setting, where it conducted history-taking and generated differential diagnoses that included the final diagnosis in 90% of cases (75% top-3 accuracy), with high patient satisfaction and no safety interventions required during monitored interactions [16,17]. These examples illustrate that AI is increasingly participating in early stages of clinical reasoning workflows, including data gathering, hypothesis generation, and preliminary synthesis, highlighting the urgency of preparing learners to critically engage with AI-generated outputs. The key educational challenge, therefore, is not whether to allow AI use, but how to teach AI literacy: the ability to critically appraise and challenge machine-generated content [18].

Instead of asking whether AI should be allowed, the more pressing question is how classroom-based learning should be redesigned to preserve its core purpose—developing clinical reasoning—in an AI-augmented environment. In practice, this requires moving beyond fact-based prompts toward reasoning-oriented tasks. For example, instead of asking “What is the most likely diagnosis?”, facilitators might ask: *“What features in the case support your leading diagnosis, and which findings argue against it?”* or *“How would your differential change if one key variable were altered?”.*

In another approach, students should be required to articulate their own differentials and justification before consulting AI, and then use AI responses to refine their reasoning [19]. For instance, in a classroom-based session, small groups may first construct and defend a differential diagnosis without AI, then generate an AI response to compare discrepancies, and finally justify a revised decision to peers. Such activities transform AI into a “debating partner” rather than a substitute for reasoning [20]. This structured sequencing helps maintain the cognitive effort essential for clinical reasoning, particularly given evidence that uncritical Generative AI use may reduce deeper engagement [6], and can be further reinforced by requiring group discussion and justification before and after AI use.

Localization and contextualization are equally important. By embedding cultural, institutional, or patient-specific details, learning sessions remain relevant to learners and less susceptible to generic AI responses [2]. For example, in a resource-limited setting, a STEMI case could specify that primary PCI is not available within the recommended timeframe. Students might be asked: *“Given this context, would you prioritize fibrinolysis with antithrombotic therapy or transfer for delayed PCI, and how do current guidelines inform your decision?”.* Such prompts require learners to adapt evidence-based recommendations to real-world constraints.

Clear classroom policies can help: policies should emphasize transparency, accountability, and assessment of students’ independent reasoning. For instance, students could be required to explicitly disclose how AI was used in their preparation and to identify which elements of their reasoning were generated independently versus AI-assisted. In addition, assessment can prioritize justification and reasoning processes over final answers, for example, grading students on how they defend their clinical decisions, critique AI-generated suggestions, or identify potential errors or biases in AI outputs. Transparency in AI use enables innovation while safeguarding academic integrity [7].

Faculty development is the final pillar [8]. Educators need training to recognize when students rely excessively on AI and to redirect attention toward reasoning. They should also be equipped with strategies to challenge AI-derived answers and probe students’ clinical thinking. Without empowered facilitators, classroom reforms will falter [18].

**Fig 1:**
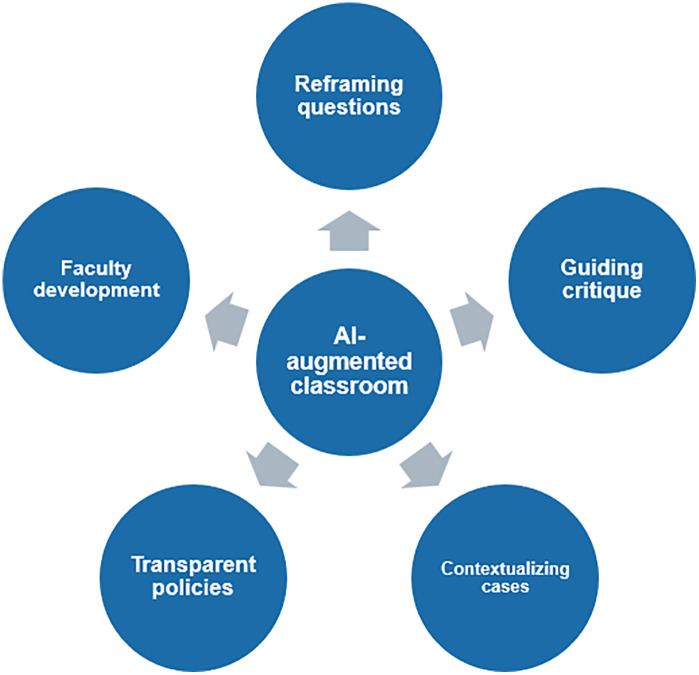
Micro-level reforms to support clinical reasoning development in AI-augmented classroom-based learning. The figure presents five complementary strategies: (1) reframing questions to prioritize reasoning-oriented prompts over factual recall; (2) guiding critique by positioning AI as a tool for interrogation and comparison; (3) contextualizing cases through the inclusion of patient-specific, institutional, and resource-related factors; (4) establishing transparent policies that emphasize accountability, disclosure of AI use, and assessment of independent reasoning; and (5) training faculty to facilitate discussion, challenge AI-derived outputs, and probe students’ clinical thinking. Together, these strategies structure classroom-based learning to support clinical reasoning in AI-augmented environments, where students articulate their reasoning, critically engage with AI outputs, and iteratively refine their judgments through peer discussion and guided facilitation in a structured, low-stakes setting.

AI in the classroom presents both opportunities and risks. If used uncritically, it risks reducing clinical reasoning to superficial pattern-matching. If guided wisely, it can challenge students to sharpen their judgment by critiquing AI rather than copying it. Our position is clear: banning AI is not the solution. Instead, educators and policymakers should embrace micro-level reforms (Fig 1): reframing questions, guiding critique, contextualizing cases, transparent policies, and training faculty. Ultimately, the goal is not to compete with AI, but to ensure that classroom-based learning continues to cultivate clinical reasoning by making students’ thinking visible, discussable, and open to challenge.
